# Transcranial Doppler in 150 Congolese children with sickle cell disease

**DOI:** 10.3389/fstro.2024.1384767

**Published:** 2024-09-18

**Authors:** Gisele Tshiama Kazadi, Didier Mukendi Mbuyi, Robert Kitenge, Smith Mpaka, Jean Lambert Ehungu Gini, René Ngiyulu, Léon Muepu Tshilolo

**Affiliations:** ^1^Department of Pediatrics, Monkole Hospital, Kinshasa, Democratic Republic of Congo; ^2^Department of Sciences and Technology, University of Kinshasa, Kinshasa, Democratic Republic of Congo; ^3^Isntitut de Recherche Biomédicale/Centre de Formation et d'Appui Sanitaire, Monkole Hospital, Kinshasa, Democratic Republic of Congo; ^4^Institut Supérieur de Statistiques de Kinshasa, Kinshasa, Democratic Republic of Congo; ^5^Department of Pediatrics, Unit of Hematology, University of Kinshasa, Kinshasa, Democratic Republic of Congo

**Keywords:** sickle cell disease, Democratic Republic of Congo, abnormal Transcranial Doppler, hydroxyurea, fetal hemoglobin, stroke

## Abstract

**Introduction:**

Sickle Cell Disease (SCD) ranks among the most prevalent genetic disorders globally. The incidence in sub-Saharan African countries has been estimated to be 230.000/y with a high prevalence (1%) in the Democratic Republic of Congo (DRC). Stroke is a significant complication of Sickle Cell Disease (SCD), and carries a high risk of disability and mortality. Transcranial Doppler (TCD) is currently the non-invasive exploration recommended for the prevention of stroke in young SCD patients.

**Objective:**

To determine the prevalence of pathological TCD in a population of young Congolese SCA patients and to assess its association with hematological parameters.

**Population and methods:**

This cross sectional study was carried out on 150 Congolese SS homozygous children between the ages 2–16 years old (mean age: 8.5 ± 4.0 years) in stable condition, and followed from January 1 to December 31, 2013. TCD was performed using the STOP I method in the main cerebral arteries. The risk of stroke was absent when the average maximum speed during a cycle (TAMMV) in middle cerebral artery (MCA) was < 170 cm/s, but present when TAMMV was borderline or conditional for values between 170 and 199 cm/s and pathological for values ≥ 200 cm/s.

**Results:**

The prevalence of pathological TCD was 4% while the conditional TCD prevalence was 10%. The Mean blood velocity in MCA was 114.0 cm/s. There was a significant difference in the means of WBC (*p* = 0.003), Hb (*p* < 0.001), Hct (*p* < 0.001), MCV (*p* = 0.005) parameters when comparing normal and at risk TCD (conditional and abnormal). However, no significant association was found for the categorical corresponding parameters

**Conclusion:**

Globally, 14% of patients were at risk of stroke, hence the interest in integrating TCD in the routine monitoring of children with SCD in order to prevent overt stroke by implementing a chronic blood transfusion program or the use of hydroxycarbamide.

## Introduction

Sickle cell disease is one of the most common genetic diseases in the world. It is widespread, with a high prevalence in tropical regions where malaria is endemic. It is estimated that around 75% of the 300,000 annual global children born with sickle cell anemia will occur in sub-Saharan Africa (SSA) (Weatherall and Clegg, 2001; Piel et al., 2023; GBD 2021 Sickle Cell Disease Collaborators, 2021). This includes the Democratic Republic of Congo (DRC) where the prevalence in newborns is around 1.4% (Tshilolo et al., 2009; Piel et al., 2013). SCD is a multisystem disease characterized by acute episodes of illness related to vaso-occlusion and infection, causing problems such as acute pain, acute chest syndrome (ACS), kidney failure, strokes and death.

The pathophysiology mechanisms of neurovascular complications and stroke in SCD are complex and not clearly understood. One leading hypothesis suggests that vascular lesions in sickle cell anemia are caused by inflammation and abnormal adhesion of sickle red blood cells (RBCs) and white blood cells (WBCs) to the vascular endothelium, leading to damage of the vascular wall (hyperplasia) (Connes et al., 2013). Post-ischemic injury, resulting from recurrent episodes of vaso-occlusion and reperfusion, is believed to play a significant role in the development of cerebral vasculopathy in SCA (Connes et al., 2013; Switzer et al., 2006). The interplay of genetic factors, sickling, subsequent ischemia, hemolysis, vaso-occlusion, and vasculopathy induced by hemoglobin S may contribute to complications of sickle cell disease, such as stroke.

Strokes can also be induced by the combined effects of genetic factors, sickling, subsequent ischemia, hemolysis, vaso-occlusion, and vasculopathy induced by hemoglobin S (Verlhac and Bernaudin, 2002).

The absence of α-thalassemia and glucose-6-phosphate dehydrogenase (G6PD) deficiency could increase the risks for stroke by modulating the level of hemolysis, oxidative stress and blood rheology (Connes et al., 2013)

Strokes are a major public health issue, representing the third leading cause of mortality and disability in high-income countries (Verlhac and Bernaudin, 2002; Adams et al., 2004; Brousse et al., 2009). Recently, GBD Collaborators (2021) reported that in 2019, strokes were also the third-leading cause of death and disability, in terms of total Disability-Adjusted Life Years (DALYs).

However, it's not only the prerogative of adults, but children are also affected by the strokes, especially when they are affected with SCD (GBD 2021 Sickle Cell Disease Collaborators, 2021; Verlhac and Bernaudin, 2002; Adams et al., 2004; Colombatti et al., 2009; Adams, 2007). Furthermore, it has been shown that SCD is the greatest contributor to mortality in children aged between 5 and 14-years of age in SSA countries (GBD 2021 Sickle Cell Disease Collaborators, 2021).

Since the work of Adams in 1992, Transcranial Doppler (TCD) has allowed the early detection of stenosis of the main cerebral arteries in children with sickle cell disease. The technique has a sensitivity of 90% and a specificity of 100% (Verlhac and Bernaudin, 2002; Roberts et al., 2009; Adams, 2005). TCD is considered normal when the time-averaged maximum of the mean velocity (TAMMV) is < 170 cm/s, conditional if 170–199 cm/s and abnormal or high risk if ≥200 cm/s (Verlhac and Bernaudin, 2002; Adams, 2007; Bernaudin, 2008).

In most sub-Saharan African countries, Transcranial Doppler (TCD) screening is not routinely conducted for children with Sickle Cell Disease (SCD). Nonetheless, a recent multicenter pilot study reported abnormal TCD findings in 0.4% of cases and a conditional risk in 17% (O'Brien et al., 2023). Higher prevalence rates have been observed in other studies: Dorie et al. (2015) reported 8.1% abnormal TCD in Mali, while Abdullahi et al. (2021) found 14% conditional TCD and 4% abnormal TCD in Nigeria. Another study documented an abnormal TCD prevalence of 10.8% among children with SCA (Lagunju et al., 2012). Overall, TCD remains largely inaccessible in many low-income countries with high SCD prevalence.

In a recent review of strokes in high and middle incomes countries, Bhattacharya et al. (2021) reported that in the Dominican Republic, SCD patients displayed 22.3% conditional and 3.9% abnormal TCD velocities, respectively. In Jamaica, Rankine-Mullings et al. (2018) reported thirteen percent and 6.7% of 358 children with SCA (mean age 7.4 ± 2.7 years), with conditional and abnormal TCD velocities, respectively. In Brazil, investigators found a low percent of abnormal and conditional TCD velocities in their cohort: 1.2 and 6.5%, respectively (Leite et al., 2012). In India, Dipty et al. (2019) reported in a TCD screening of 120 children, 4.2% of abnormal TAMMV, 6.7% of conditional TAMMV, and 11.7% of unobtainable or low velocities.

To our knowledge, there is little data on TCD reported in Congolese SCD children (O'Brien et al., 2023). We therefore performed this pilot study on TCD in 150 Congolese SCA children in order to determine the prevalence of risks of stroke and the related hematological parameters.

## Population and methods

Participants were selected among sickle cell patients regularly treated in Monkole Hospital, a second level General Hospital with a platform for screening and monitoring children with sickle cell disease. One hundred and fifty (150) Congolese SS homozygous children aged 2–16 years, in steady-state, were selected, from January 1 to December 31, 2013. The sample was calculated according to the formula *N* = ${\text{Z}}_{\alpha }^{2}$ *p* (1–*p*)/*d*^2^ where the proportion *p* of SCD children at risk of stroke was set at 10% in accordance with the estimated value in most of the recent papers (Adams, 2007; O'Brien et al., 2023; Abdullahi et al., 2021) Z_α_ = 1.96 and *d* = 5%. A minimum sample size of 139 patients was obtained.

All patients received a confirmatory test for SCD using capillary electrophoresis with a Sebia 2 CN 20403.05 machine. None of the recruited patients were transfused during the 3 months prior to entering the study, and none were on hydroxyurea. Folic acid was administered to all patients, and oral penicillin was given to those under 5 years of age. All the patients had no history of previous seizures, diabetes, hypertension and HIV infection when included in this study.

This study was approved by the Research Ethics Committee of the Public Health School of the University of Kinshasa, DR Congo, ESP/CE/53/023. Informed consent was obtained from the patient's responsible guardians before their participation in the study. Health workers (nurses and physicians) in Monkole Hospital have been previously educated and well-trained in the use of a non-imaging transcranial doppler with a probe of frequency 2 Mhz (ATys BD Doppler echography, S/N Model, Wakie 230080). Explorations were performed by two physicians well-trained using the STOP method (Roberts et al., 2009). TCD was performed when patients were in steady state, without any complication. Measurements were taken through the temporal and occipital windows in quiet children. TCD was used by measuring the speed of blood flow in the cerebral arteries, exploring the middle cerebral arteries (MCA), and the anterior cerebral arteries (ACA). TCD was considered normal when the time-averaged maximum of the mean velocity (TAMMV) was between 50 and < 170 cm/s, conditional if 170–199 cm/s and abnormal or high risk if ≥200 cm/s (Verlhac and Bernaudin, 2002; Bernaudin, 2008; O'Brien et al., 2023).

Blood parameters (Blood Cell counts and the Wintrobe indices) were measured at the time of each TCD examination with a Sysmex XS−1000i machine and Fetal hemoglobin (HbF) was checked by capillary electrophoresis with a Capillarys 2 System Sebia CN 20403.05.

The blood flow measurements were correlated with demographic data and blood parameters.

### Statistical analysis

Data was then imported into SPSS software (IBM SPSS version 20), for statistical analysis. Continuous variables were summarized using means, standard deviations, and quantiles. As the normality hypothesis was not met, a Wilcoxon signed test was used to compare outcomes in paired samples, such as when comparing TAMMV in the right vs. in the left. For independent samples, we used the Wilcoxon rank-sum test. Moreover, logistic regression was used to assess the dependence of hematological parameters on the binary outcomes. A significant level of 5% was used for all statistical tests.

## Results

One hundred and fifty homozygous sickle cell patients (SS) were recruited in the study, with a sex ratio 1.1 and a mean age 8.4 ± 3.9 years (range 2–16 yrs.); the mean Hb was 7.3 with a range of 3.6 g−9.9 g/dl. The one with Hb values of 3.6 g/dl was a 5 years old girl with GB ≥ 15,000, Plt = 129,000 and TAMMV in MCA of 211 cm/s. There was significant difference on the means of Hb between patients with normal TCD vs. the others; those with normal TCD had higher value. Moreover, the difference was not significant when comparing patients with abnormal TCD (mean Hb 7.5 g/dl) and conditional TCD (mean Hb= 7.7 g/dl; *p* = 0.73).

Demographic data and hematological parameters are reported in Table 1.

**Table 1:** Demographic and hematological parameters in Congolese children with sickle cell anemia.

	**Mean**	**SD**	**Min**	**Q25**	**Median**	**Q75**	**Max**
**Clinical data**							
Age (years)	8.48	3.95	2.04	5.13	8.01	11.7	16.11
Weigh (kg)	24.1	10.4	9	16	22	29.3	61
Height (cm)	125.2	23.38	1.4	110.7	127	141	171.8
BMI	14.52	2.15	10.4	13.2	14.1	15.7	21.9
Nb of transfusions	3.08	3.1	0	1	2	4	20
ASS	0.33	0.69	0	0	0	0	3
ACS/yr	0.28	0.67	0	0	0	0	5
Nb of VOC/yr	1.75	1.92	0	0	1	3	10
Nb of hospitalization/yr	0.93	1.41	0	0	1	1	12
**Hematological parameters**							
Age (years)	8.4	3.9	2.0	5.1	8.1	11.7	16.1
WBC (G/L)	14.0	4.5	3.6	1.1	13.5	16.8	28.9
Hb (g/dl)	7.3	1.0	3.6	6.7	7.4	7.9	9.9
HCT (%)	21.7	3.2	13.0	19.5	21.8	23.5	30.4
PLT (G/L)	403.3.	173.5	109.	293.5	389.5	476.0	998.0
MCV (fl)	78.3	10.7	53.0	71.7	78.0	84.5	112.1
HbF (%)	4.8	3.1	0	2.5	4.2	6.9	16.3

BMI, body mass index; Nb, number; ASS, acute splenic sequestration; ACS, acute chest syndrome; VOC, vaso-occlusive crisis; Yr, year; WBC, white blood cells; Hb, hemoglobin; Hct, hematocrit; PLT, platelets; MCV, mean corpuscular volume; HbF, fetal hemoglobin.

Globally, the mean TAMMV in MCA was 114 cm/s (range: 43–211). No significant difference was found when comparing the average values between the left and right sides.

The blood flow velocity (TAMMV) measured in the MCA (right and left) was normal in 129 patients (86%), conditional in 15 patients (10%) and abnormal in six patients (4%) (Table 2). Among the patients observed, three individuals (2%) exhibited a reduced velocity of < 50 cm/s in the MCA arteries. These cases involved two females, aged 3 and 14, with corresponding HbF values of 3.6 and 3.5%, respectively, and a 16-year-old male with an HbF value of 8.9%. Notably, all three patients also exhibited low levels of hemoglobin, measuring < 7 g/dl.

**Table 2:** TCD assessment of TAMMV values in MCA related to age group.

**Age (years)**	**Normal**	**Conditional**	**Abnormal**	**Total (*n*)**
	<**170 cm/s**	**170–199 cm/s**	> **200 cm/s**	
< 5 yrs	29 (20%)	3 (2%)	2 (1.3%)	34 (24%)
5–10 yrs	50 (33.3%)	7 (4.7%)	3 (2%)	60 (40%)
>11–15 yrs	35 (23.3%)	5 (3.3%)	1 (1.3%)	41 (28%)
>15 yrs	11 (7.3%)	0	0	11 (8%)
Total	125 (84%)	15 (10%)	6 (4%)	146 (100%)

MCA, middle cerebral artery.

Abnormal and Conditional TCD were prevalent in children aged < 10 years (*n*15/21). Three patients aged 3, 14, and 16 displayed low values < 50 cm/s.

Related to the age group, abnormal and conditional TCD were prevalent in children aged < 10 years (*n*15/21) (Table 2).

Patients with conditional and abnormal TCD displayed higher values of WBC, lower Hb and HbF when compared to those with normal TCD.

There was a significant difference in the means of WBC (*p* = 0.003), Hb (*p* < 0.001), Hct (*p* < 0.001), MCV (*p* = 0.005) parameters when comparing normal and no normal TCD. However, no significant association was found for the categorical corresponding parameters.

## Discussion

TCD is a non-invasive exploration for the prevention of stroke in sickle cell patients (Adams, 2007; Bhattacharya et al., 2021). The regular use of TCD reduced the incidence of ischemic stroke from 11 to 1% with monitoring, providing the opportunity for preventive therapeutic interventions (Adams, 2007). Strokes are a major complications in young children under the age 5, it is 0.4% at 19 years old (Adams et al., 1992). Children with 2–9 years old are more at risk, because this is the period age of onset of endothelial intimal hyperplasia and then indicated for initiating preventive measures (Verlhac and Bernaudin, 2002; Roberts et al., 2009; O'Brien et al., 2023). In this study, we observed that the age group with the highest median of TAMMV was in groups aged < 10 years (Table 2). This trend was in agreement with the report by Lagunju et al. (2012) and Makani et al. (2009) among patients with HbSS genotype.

In this study, the global prevalence of abnormal and conditional Transcranial Doppler (TCD) was of 14%, with 6 patients (4%) showing abnormal TCD and 15 (10%) exhibiting conditional TCD. These rates are somewhat comparable to and differ from those reported by other researchers in Africa. In Nigeria, Abdullahi et al. (2021) and Lagunju et al. (2012) reported a 4% prevalence of abnormal TCD, whereas Prussien et al. (2019) and Modebe et al. (2023) found higher rates of 7.2 and 6%, respectively. Soyebi et al. (2014), in a large cohort of 2300 Nigerian SCD patients, documented 9.4% prevalence of abnormal TCD and 19.3% for conditional TCD. The discrepancies in prevalence within the same country likely result from variations in screening methods and the number of patients included in each study. In another West African country, Mali, Dorie et al. (2015) reported a prevalence of 8.1% abnormal TCD and 17.3% conditional TCD in homozygous SS patients.

In contrast, other studies realized in the Central and East part of Africa, reported lower prevalence of abnormal TCD than those reported in Nigeria: In Tanzania, Kija et al. (2019) and Ambrose et al. (2020) reported a prevalence of 0.5 and 2% while Makani et al. in Kenya reported in a cohort of 105 patients no case of abnormal TCD. In a recent study in Sub Saharan African countries (DRC, Zambia and Malawi) (*n* = 770) O'Brien et al. (2023) reported a very low prevalence of abnormal TCD (0.4%) but with a higher prevalence of conditional TCD (17%). These contrasting results could be due to the variability of methods and selection of patients but also to possible genetic and environmental factors linked to different geographical areas. In fact, it's well-known that various genetic modulators can affect the clinical expression of SCD.

Differences could be associated with Bantu haplotype which is associated with a high rate of alpha chain deletion (Nagel, 2001; Mouele et al., 2000; Ojewunmi et al., 2021). Alpha thalassemia is a modulator of Hb S that decreases total hemoglobin value, hemolytic markers, including the number of leukocytes and hemoglobin S concentration. The low value is supposed to reduce sickling and hemolysis (Bernaudin et al., 2011). Elsewhere, Ojewunmi et al. (2021) demonstrated in their Nigerian cohort that 48/67 (69.6%) of patients with abnormal TCD were without alpha thalassemia. The absence of a-thalassaemia and glucose-6-phosphate dehydrogenase (G6PD) deficiency could increase the risks for stroke by modulating the level of hemolysis, oxidative stress and blood rheology (Connes et al., 2013). The high frequency of alpha thalassemia in Congolese and patients bearing bantu haplotype could explain why the prevalence of abnormal TCD is relatively low when compared to data in West Africa (O'Brien et al., 2023; Prussien et al., 2019; Modebe et al., 2023; Soyebi et al., 2014) in USA and Europe (Adams, 2007; Bernaudin et al., 2011).

On the other hand, strokes were also reported in patients with low values of TAMMV in MCA or ACA. Buchanan et al. (2013) described five cases of stroke in SCA patients with TCD flow velocity < 70 cm/s and who developed an overt cerebral infarction, silent stroke or transient ischemic attack. They suggest that TCD velocities < 70 cm/s in major vessels (MCA, ACA, and ICA) be considered another type of “abnormal,” prompting more sensitive evaluations (such as a brain MRI and MRA) for the presence of central nervous system disease, and, if negative, decrease intervals between subsequent TCD assessments (Buchanan et al., 2013).

Elsewhere, Kija et al. (2019), had found during their study 20% of patients with blood flow velocities below 50 mm/s. In 49 out of 67 children with low/absent/elevated transcranial doppler undergoing magnetic resonance imaging, 43% had infarction, whereas 24 out of 48 (50%) magnetic resonance angiographies were abnormal.

Contrary to the 70 cm/s cut-off used by Buchanan et al. (2013), the study defined low TCD values using a 50 cm/s cut-off, as utilized by Kija et al. (2019) and O'Brien et al. (2023). Three cases were identified (2%) with low TCD, although the prevalence would likely be higher if the 70 cm/s cut-off were applied. In a cohort of 770 African SCD children from the DRC, Zambia, and Malawi, O'Brien et al. reported a 4% prevalence of low TCD values. This prevalence was 19% in a Tanzanian cohort (*n* = 200) (Kija et al., 2019) and 7% in a cohort of 105 patients in Kenya (Makani et al., 2009). In a study of 120 SCD patients, Dipty et al. (2019) identified 12 cases (10%) with low TCD values, including two with stroke and one with seizures. These findings highlight the necessity of including patients with low TCD values among the “abnormal” or high-risk categories (Buchanan et al., 2013). Notably, paradoxical reductions in TCD flow velocities may occur in arteries with long segments of stenosis or in cases of >50% stenosis in multiple arteries, classified as “diffuse intracranial disease,” such as Moyamoya or extracranial vasculopathy (Buchanan et al., 2013; DeBaun and Kirkham, 2016). Further investigations with MRI are recommended to diagnose silent infarcts or other vasculopathies associated with neurological symptoms like headaches, seizures, or cognitive disorders (Bernaudin et al., 2011; Buchanan et al., 2013; DeBaun and Kirkham, 2016). However, in our study, we were unable to conduct extended exploration with magnetic resonance angiography.

Concerning the hematological parameters, a level of Hb < 6 g/dl, WBC values ≥ 15,000 cells/dl and MCV ≥ 85 fl were found to be risk factors for sickle cell patients. The low Hb level was correlated in the present study with pathological TCD (*p* < 0.001). This observation corroborates with what recorded by other authors (Lagunju et al., 2012; Rankine-Mullings et al., 2018; Hokazono et al., 2011; Chukwu et al., 2021). Adams and Nichols (1990) had described that hypoxia induced by severe anemia increases blood flow velocity and could end in the presence of already established vascular intima hyperplasia lead to stroke.

The present study did not show any association between the low Hb F value and the pathological TCD (*p* = 0.27) when taking in account for the other variables. Significant association was reported by other studies which had also reported a positive correlation: Adams and Nichols (1990) and Hokazono et al. (2011) (*p* = 0.03), Gulbis et al. (2005) (*p* < 0.01), Bernaudin et al. (2005) (*p* < 0.01) as well as Chukwu et al. (2021) (*p* = 0.007). This correlation is due to the inhibition of hemoglobin S polymerization and endothelial adhesion of erythrocytes. Hb F slightly increases the overall concentration of total Hb (alleviates anemia) and MCV, and reduces the production of neutrophils, in short it improves rheology. The positive impact of hydroxycarbamide in reducing the overt stroke risk is mainly due to the increase HbF (Tshilolo et al., 2019; Di Mauro et al., 2023). In accordance with our results, Ojewunmi et al. (2021) (*p* = 0.294) and Cox et al. (2014) (*p* = 0.672), did not found in their studies an association between low Hb F and pathological TCD. These discrepancies between these different studies on the role of HbF on the overt stroke need further multinational studies on large cohorts of SCA patients coupled to genetic markers and other parameters like G6PD, alpha thalassemia deletions and Hb saturation which can play a role in the complex physiopathology of stroke in SCD (Adams, 2007; Bernaudin, 2008).

The study noted a protective effect of the MCV rate < 85 fl (*p* < 0.01) OR 95%: 5.23 (1.51–18.13). This result corroborates the results reported by Bernaudin et al. (*p* < 0.01) (Pincez and Lettre, 2023), Ojewunmi et al. (2021) (*p* < 0.001), and Rankine-Mullings et al. (2018) (*p* = 0.005). The decrease in hemoglobin due to possible co-heritance of α thalassemia, has been a marker that would protect against the elevation of cerebral blood flow velocities. This correlation is induced by the protective action of α thalassemia whose prevalence in Central Africa varies around 25–40% (Mouele et al., 2000; Ohene-Frempong et al., 1998).

The mean value of Hb cohort was 7.3 ± 1 g/dl and no significant difference was observed when compared to the patients with abnormal or conditional TCD who displayed values of 5.7 g/dl and 6.7, respectively. The relative low prevalence of abnormal and conditional TCD in the series compared to what described in patients living in USA or Europe (Adams and Nichols, 1990; Bernaudin et al., 2005) could be due to the low level of Hb as some authors reported that hypoxia induced by severe anemia increases blood flow velocity (Adams, 2007; Gulbis et al., 2005). Elsewhere, Ohene-Frempong et al. (1998) demonstrated that low steady-state Hb level was an independent risk factor for ischemic stroke and that hemorrhagic stroke was associated with low Hb and high leukocyte count. Adekunle et al. (2017) in Lagos, Nigeria also observed a significant correlation between TAMMV and leukocytosis and this could be ascribed to the fact that these cells are active participants in the chronic inflammatory process in SCD. Our patients displayed both low hemoglobin and high WBC count but no so high prevalence of abnormal TCD as reported in Nigeria. We did not check for the presence of G6PD in our series. Conflicting reports of an effect of G6PD on cerebral brain flow velocities (Bernaudin et al., 2008; Miller et al., 2011) may result from variations between populations in either the phenotypic expression compared to assessed genotype or from methods of study. Finally, the low prevalence of stroke reported in most of the African countries would also be due to the lack of newborn screening and the high mortality in young SCA children who died without a diagnosis of SCA or early in childhood before vasculopathy developed (Therrell et al., 2020).

Advocacy is needed for a systematic screening of abnormal and conditional TCD in SCA children aged < 10 years in order to implement a preventive program as soon as possible. TCDs are portable and non-invasive, but limitations include high operator-dependence, inability to detect velocities in all patients, particularly those with poor bone windows, a low specificity for stroke, and they do not predict silent ischemia. Health care workers (doctors as nurses) well-trained are able to realize correctly such explorations.

Chronic Blood Transfusion and Cell Stem Cell transplant are the usual preventive measures used in high income countries (Adams et al., 2004; Bernaudin, 2008; O'Brien et al., 2023; Bernaudin et al., 2011; DeBaun and Kirkham, 2016; Gulbis et al., 2005; Di Mauro et al., 2023). In low income countries, hydroxyurea can be an alternative solution that can substitute blood transfusion therapy that is not routinely available (DeBaun and Kirkham, 2016). Recent studies emphasized the role of Hydroxyurea in preventing strokes in SCD patients (Pincez and Lettre, 2023; Di Mauro et al., 2023; Soulié et al., 2023).

The study has several limitations: it was not randomized but rather a pilot implementation initiative of stroke prevention using the STOP program; a large cohort study would be interesting to monitor the impact of variation in the blood flow velocities. All the TCD explorations were performed using the non-imaging transcranial Doppler that can be less performant in comparison with the imaging TCD. Supplementary IRM was not available for the precision of patients with very low TCD values that could be due to severe stenosis or limited window exploration.

## Conclusion

This study displayed a prevalence of pathological TCD similar to what observed in some other African studies and highlighted the influence of some hematological parameters (MCV, low Hb, low HbF).

In spite of the relative low frequency of abnormal TCD (4%) observed in this study (when comparing to other African countries,) a systematic TCD is indicated in young children and we hope that this preliminary study will contribute to the implementing of a routine screening of stroke in patients living in DRC and then the introduction of preventive measures based on the use of hydroxyurea where chronic blood transfusion is not available.

## Data Availability

The raw data supporting the conclusions of this article will be made available by the authors, without undue reservation.
